# Formulation of tizanidine hydrochloride–loaded provesicular system for improved oral delivery and therapeutic activity employing a 2^3^ full factorial design

**DOI:** 10.1007/s13346-022-01217-3

**Published:** 2022-08-04

**Authors:** Amira Mohamed Mohsen, Hadeer Ahmed El-Hashemy, Abeer Salama, Asmaa Badawy Darwish

**Affiliations:** 1 Pharmaceutical Technology Department, National Research Centre, El-Buhouth St., Dokki, Cairo, 12622 Egypt; 2 Pharmacology Department, National Research Centre, El-Buhouth St., Dokki, Cairo, 12622 Egypt

**Keywords:** Tizanidine, Proniosomes, Full factorial design, Muscle relaxant, GABA, EAAT2

## Abstract

Tizanidine hydrochloride (TZN) is one of the most effective centrally acting skeletal muscle relaxants. The objective of this study is to prepare TZN-loaded proniosomes (TZN-PN) aiming at enhanced oral delivery and therapeutic activity. TZN-PN were prepared by coacervation phase separation method. The developed vesicles were characterized via entrapment efficiency percentage (EE%), vesicular size (VS), and zeta potential (ZP). A 2^3^ full factorial design was employed to attain an optimized TZN-PN formulation. The optimized TZN-PN were further characterized via in vitro release study and transmission electron microscopy (TEM). In vivo rotarod test was employed for determination of the muscle relaxant activities of rats and levels of GABA and EAAT2 were detected. The developed TZN-PN exhibited relatively high EE% (75.78–85.45%), a VS ranging between (348–559 nm), and a ZP (−26.47 to −59.64). In vitro release profiles revealed sustained release of TZN from the optimized TZN-PN, compared to free drug up to 24 h. In vivo rotarod study revealed that the elevation in coordination was in the following order: normal control < free TZN < market product < TZN-PN (F6). Moreover, the optimized TZN-PN exhibited significant elevated coordination activity by 39% and 26% compared to control group and market product group, respectively. This was accompanied with an elevation in both GABA and EAAT2 serum levels. Thus, it could be concluded that encapsulation of TZN in the provesicular nanosystem proniosomes has enhanced the anti-nociceptive effect of the drug and consequently its therapeutic activity.

**Graphical abstract:**

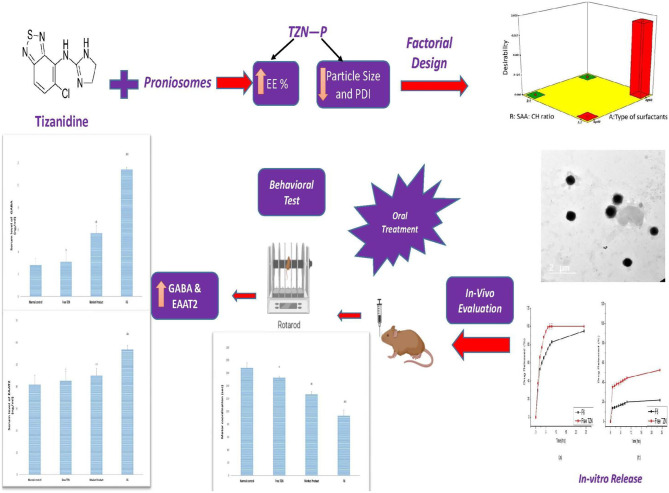

## Introduction

Spasticity can be defined as a motor disease characterized by a velocity-dependent increase in tonic stretch reflexes and excessive tendon jerks [1]. The source of spasticity is uncertain; however, it is believed to be caused by excess activation or lack of inhibition of motor neurons, resulting in higher muscle tone. Multiple sclerosis, cerebral palsy, spinal cord damage, traumatic brain injury, and post-stroke syndrome are all causes of spasticity [2]. Antispasticity treatments decrease muscular tone via affecting the central nervous system (CNS) or the skeletal muscles directly. Baclofen, tizanidine, gabapentinoids, pregabalin, riluzole, and benzodiazepines are CNS agents, while dantrolene and botulinum toxin are peripheral agents [3–5].

Tizanidine hydrochloride (TZN) is one of the most effective centrally acting skeletal muscle relaxants. TZN is an α_2_-adrenergic receptor agonist that reduces spasticity by enhancing motor neuron presynaptic inhibition. It works mainly at the level of the spinal cord and is used to treat spasticity caused by spinal cord damage, illnesses, or multiple sclerosis [6]. TZN is used to treat painful muscle spasms and spasticity in conditions like chronic headaches, back pain, and postoperative pain. TZN is a BCS class II medication with high permeability and low solubility. Because of the drug’s extensive first-pass metabolic action, it has a limited oral bioavailability (about 21% of the drug is only available in the blood circulation). Furthermore, its average elimination half-life is about 3 h [7–9]. Thus, it must be given to patients on a regular basis to sustain the therapeutic impact. Consequently, it is crucial to develop a new TZN delivery strategy that can improve its oral delivery and pharmacological activity while minimizing its side effects.

Over the last few decades, several drug delivery systems have been developed and evaluated in order to develop an efficient, effective therapeutic treatment that can overcome the problems associated with traditional drug delivery systems, such as poor patient compliance, hepatic first-pass metabolism, undesirable side effects, and rejection of invasive treatments. A lot of efforts have focused incorporating the drugs in a nanosystem in order to improve their efficacy and safety [10]. Nanotechnology has aided scientists in thinking more clearly and developing more effective treatments [11]. Various nanosystems have been designed to encapsulate various types of pharmaceuticals aiming at enhancing their activity and bioavailability [12–14].

The vesicular systems were expected to increase the duration of the medication in systemic circulation and reduce toxicity by selective uptake together with lymphatic transport, resulting in better penetration as well as rate and extent of absorption by avoiding first pass metabolism [15]. A recent contemporary technology to develop vesicular systems, the provesicular system, was presented without changing or influencing intrinsic features of the pharmaceuticals entrapped in order to enhance the stability of the vesicular systems [16].

Provesicular systems [17], such as proniosomes, are dry, free-flowing formulations of a surfactant-coated carrier [18]. Proniosomes are made up of the standard vesicular components as well as ethanol and small amount of water [19]. The development of a vesicle “concentration” in the preparation of proniosomes is dependent on lowering the water content present in niosomal formulations [20]. The majority of research studies that have investigated the behavior of niosomes (hydrated product of proniosomes) have found that the drug’s pharmacokinetics were significantly changed after being encapsulated in the vesicular system. Proniosomes have been demonstrated to show higher drug solubility, resulting in improved drug absorption [21]. Several studies have reported the ability of proniosomes to improve the absorption and hence the bioavailability of drugs via the oral route [22, 23].

Proniosomes are biodegradable, biocompatible, and non-immunogenic carriers with high chemical and physical stability that facilitate their handling and storage [21]. Proniosomes can improve the bioavailability of both hydrophilic and lipophilic drugs. They have the ability to deliver medications efficiently via several channels to precise sites of action. Proniosomes have been investigated via several routes of administration such as oral, parenteral, transdermal, ophthalmic, oral mucosal, vaginal, pulmonary, and intranasal [22–25]. They have the potential to boost pharmacological efficacy, lessen or eliminate drug side effects, and increase its therapeutic action. Furthermore, proniosomes help to keep drug levels at therapeutic levels for longer, reduce administration frequency, and enhance patient compliance [26].

The aim of this study is to prepare and assess TZN-loaded proniosomes aiming at enhancing the oral delivery and therapeutic efficacy of TZN. A full factorial experimental design was employed to investigate the effects of numerous factors (type and concentration of edge activators) on the studied responses (entrapment efficiency percentage (EE %), vesicular size (VS), and zeta potential (ZP)) in order to reach optimal characteristics. Factorial designs are well known in pharmaceutical research that emphasizes the effects of formulation variables and their interactions on response variables as when they are properly designed, they produce the best information from the fewest investigations [27, 28]. The in vitro drug release and in vivo efficacy of the optimized formulation were examined to be compared with free TZN and market product.

## Materials and methods

### Materials

Tizanidine hydrochloride (TZ) was generously offered as a gift by Hi pharm Co., Cairo, Egypt. Cholesterol (CH) (95%) was supplied by Panreac (Barcelona, Spain). Span 40^®^ (sorbitan monopalmitate) (Sp40) and Span 60^®^ (sorbitan monostearate) (Sp60) were procured from Sigma-Aldrich (St. Louis, MO). Gamma aminobutyric acid (GABA) and excitatory amino acid transporter 2 (EAAT2) enzyme-linked immunosorbent assay (ELISA) kits were procured from NOVA kits (Beijing, China). All other chemicals were of analytical grade.

### Animals

Albino male Wistar rats weighing 120 to 140 g were procured from the animal house of the National Research Centre (NRC; Cairo, Egypt). The animals were treated humanely according to the NRC’s animal care and use committee’s rules. During the research period, the animals were given free access to regular food pellets and water. Experiments were accomplished according to the principles of the Declaration of Helsinki. Approval was granted by the Ethical Committee of the NRC Medical Research, Dokki, Cairo, Egypt (number: 9449102021).

### Full factorial design

Design-Expert^®^ 8.0 (Design-Expert^®^, version 8.0, Stat-Ease Inc. Minneapolis, MN) was employed using full factorial design approach in the present study. Statistical design was performed with 3 factors each to be evaluated on 2 different levels with a total of 8 runs. The type of surfactants (X1), SAA:CH ratio (X2), and TZN amount (X3) were assigned to be the independent parameters. The selected dependent responses are *Y*1, entrapment efficiency “EE %”; *Y*2, vesicular size “VS”; and *Y*3, zeta potential “ZP” as shown in Table 1.

**Table 1:** Composition and characterization parameters of the developed TZN-PN

**Formulation**	**SAA type**	**SAA:CH** **molar ratio**	**TZN (mg)**	**EE (%) ± S.D**	**PS (nm) ± S.D**	**PDI**	**ZP (mV) ± S.D**
F 1	Sp40	1:1		79.42 ± 0.27	412.48 ± 4.35	0.456	−47.66 ± 1.62
F 2	Sp60		10	80.83 ± 0.33	359.62 ± 6.18	0.443	−51.37 ± 0.17
F 3	Sp40	2:1		75.78 ± 0.61	407.18 ± 2.57	0.457	−42.38 ± 0.25
F 4	Sp60			80.84 ± 0.36	348.94 ± 9.84	0.374	−55.36 ± 0.48
F 5	Sp40	1:1		82.42 ± 0.51	559.82 ± 3.57	0.416	−41.32 ± 0.59
F 6	Sp60		20	85.45 ± 0.47	348.22 ± 4.27	0.475	−59.64 ± 0.33
F 7	Sp40	2:1		81.05 ± 1.6	463.09 ± 4.65	0.374	−26.47 ± 3.92
F 8	Sp60			81.98 ± 0.92	379.14 ± 1.93	0.467	−48.89 ± 0.15

Data were displayed as mean ± S.D. (*n* = 3)

### Preparation of TZN-loaded proniosomes (TZN-PN)

TZN-PN were formulated by coacervation phase separation method described by Mishra et al. [29] with slight modifications. Briefly, in wide-mouth glass vials, precisely weighed amount of surface-active agent (SAA) either Sp40 or Sp60 along with CH was allowed to dissolve in 0.5 ml organic phase (ethanol). The vial was closed tightly and warmed for 10 min in a water bath at 60–65 °C, shaking constantly, until all of the components were thoroughly dissolved. The drug was dissolved in 0.2 ml distilled water that had been warmed to 60–65 °C. The aqueous phase was added to organic phase, which was then warmed in a water bath for another 5 min until a translucent solution was formed. After allowing the mixture to settle to ambient temperature for 24 h, the production of a yellowish white creamy gel was noticed.

#### Development of vesicles from proniosomal gels

Hydration of the produced proniosomal gels yielded niosomal vesicles. Around 10 ml of heated distilled water (70–75 °C) was added to the vial. Then, the vial was heated in a water bath at 60 °C for 10 min with intermittent mixing using a vortex mixer [30]. The formed vesicular dispersion was employed for further characterizations.

### Characterization of TZN-PN

#### Entrapment efficiency percentage (EE %)

Cooling centrifugation was used to separate the free drug from the hydrated vesicular dispersions. One milliliter of the dispersion was centrifuged at 5200 × g and 4 °C for 45 min employing a cooling centrifuge (Union 32R, Hanil, Korea). The separated pellet was washed twice by resuspending it in 10 ml distilled water and further centrifugation was performed at the same conditions. The supernatant was collected, filtered using Millipore membrane filter (0.22 µm), and measured spectrophotometrically employing a UV-spectrophotometer (Shimadzu, model UV- 2401 PC, Kyoto, Japan) at a wavelength of 319 nm [31], at a preconstructed calibration curve (*R*^2^ = 0.998, *n* = 3). The EE % was estimated by subtracting the quantity of free drug from the total TZN firstly added, using the following equation:

$$\mathrm{EE\ \%}=\frac{\mathrm{Total\ amount\ of\ TZN}-\mathrm{Free\ TZN}}{\mathrm{Total\ amount\ of\ TZN}}\times 100$$

#### Vesicular size (VS), zeta potential (ZP), and polydispersity index (PDI) analysis

The mean size of the prepared vesicles, size distribution (PDI), and ZP were investigated by photon correlation spectroscopy employing a Zeta-sizer (Nano Series ZS90, Malvern Instruments, Ltd., UK) through a helium–neon laser with a wavelength of 633 nm at room temperature. The prepared samples were diluted properly at 1:100 (v/v) with distilled water prior to measurement. The PDI is used to determine the size distribution’s width. All measurements were carried in triplicates.

### Optimization of TZN- PN

Design-Expert^®^ (8.0) software was manipulated to choose the optimal formulation by relating the desirability function. The optimization process was conducted to obtain the best formula with the highest EE % (*Y*_1_), the lowest value for VS (nm) (*Y*_2_), and the highest value of ZP (*Y*_3_) (Table 1). The solution with desirability value near to 1 was selected. For confirming the model efficiency, the selected formulation was prepared, characterized, and compared with the predicted responses. The results were statistically analyzed by one way analysis of variance (ANOVA) using SPSS^®^ software 17.0.

### Characterization of the optimized TZN-PN

#### In vitro release studies

In vitro release profiles of TZN from the optimized formula compared to free drug solution was performed employing the membrane dialysis bag diffusion technique [25]. Release studies were studied in media relevant to oral drug delivery, namely, 0.1 M HCl (pH 1.2) and PBS with a pH of 7.4 [32, 33]. A presoaked semi-permeable membrane of molecular weight (cutoff 12,000–14,000 Da) was used as a dialysis bag. The dialysis bag was filled with an amount of TZN equivalent to 2 mg and sealed on both ends. The dialysis bags were then put in 50 ml of 0.1 M HCl pH 1.2 to simulate acidic gastric media or phosphate buffer saline (PBS) pH = 7.4 to simulate the intestinal media [34, 35]. They were kept at 37 °C with a rotation speed of 100 rpm. Five-milliliter aliquots were withdrawn and replaced with an equivalent volume of fresh media at pre-determined time intervals of 1, 2, 3, 4, 5, 6, 7, 8, and 24 h. The drug concentration in the withdrawn samples was then measured spectrophotometrically at 319 nm. A plot of the cumulative percentages of drug released at each time interval (Q) and time (h) was used to create the release profile. All measurements were done in triplicates, and the mean and standard deviation were calculated.

#### Kinetics study of the release profiles

The data from the in vitro release profiles of TZN from the prepared vesicular formulations was analyzed using various mathematical models. These models comprise zero-order kinetics (cumulative percentage drug released versus time), first-order kinetics (log percentage drug retained versus time) [36], Higuchi model (cumulative percentage drug released versus square root of time) [37], and Peppas model (log cumulative percentage drug released versus log time). The release exponent “*n*” was computed in Peppas model, which indicates the drug release process [38].

#### Transmission electron microscopy (TEM)

A TEM (model JEM-1230, JEOL, Tokyo, Japan) was used to analyze the morphology of the selected TZN-PN. A drop of TZN-PN was dropped onto a carbon-coated copper grid and allowed to adhere for roughly 1 min. To act as a negative staining agent, a drop of 1% phosphotungestic acid solution was applied, and the additional solution was collected with a tip of filter paper. After staining, samples were then left for 10 min for complete dryness before being investigated.

### In vivo studies

#### Experimental design

The biological assessment of muscle relaxation activity of the selected formulations was performed on male Wistar albino rats. Thirty-two rats were randomly distributed into four groups (*n* = 8). Rats of group I received oral saline and acted as normal control. Group II received free TZN (4 mg/kg) [39] single dose orally. Groups 3 and 4 were treated with an oral single dose of market product and optimized TZN-PN formulation, respectively.

#### Muscle relaxant activity of TZN-loaded proniosomes using accelerating rotarod

Using an accelerating rotarod (Ugo Basile, Varese, Italy, model 7750), the muscle relaxation activity of free TZN, market product, and TZN-PN-optimized formulation was evaluated [40]. Rats were first trained by exposing them to a stationary rod for 5 min, followed by three sessions on the rotarod apparatus on 3 consecutive days, while gradually increasing the speed from 4 to 40 rpm over 300 s. After the training stage, rats were then allowed to stand on rotarod that began rotation at a speed of 4 rpm followed by a gradual increase to 40 rpm over 300 s. The performance of rats on rotarod was measured as the mean time spent on the rod in seconds [41]. The time lapse between the oral dosage administration and the rotarod experiment was 60 min. After performing the rotarod experiment, the blood was withdrawn from the retro-orbital vein for biochemical analysis studies.

#### Biochemical analysis of GABA and EAAT2

After rotarod test, blood samples were taken from rats of all groups via retro-orbital vein from different groups under anesthesia [42]. After allowing the blood to coagulate, it was centrifuged at 3000 rpm at 4 °C for 15 min [43]. GABA and EAAT2 levels were assessed in serum using specific ELISA kits. The manufacturer’s instructions for determining the results were performed. Standards and samples were pipetted into wells containing immobilized antibodies specific for rat GABA and EAAT2, followed by the addition of biotinylated antirat antibody. The wells were pipetted with horseradish peroxidase–conjugated streptavidin and incubated for 1 h. After 5 washes, chromogenic A and B were added to the wells. This was followed by the development of a color which was related to the amount of GABA and EAAT2 bound. The development of the color was stopped (stop solution), and the strength of the color was estimated at 450 nm [44].

### Statistical analysis

Experiments were accomplished in triplicates and results were presented as a mean ± SD. The results were statistically analyzed using the SPSS^®^ software, which performed one-way analysis of variance (ANOVA) followed by the least-significant difference test (LSD). When the *p* values were less than 0.05, the differences were considered statistically significant.

## Results and discussion

### Preparation of TZN-PN

TZN-PN were successfully developed and appeared as a yellowish transparent gel which on hydration with hot water yielded TZN-loaded niosomal dispersions. These dispersions were characterized employing several methods of characterizations, where full factorial design was applied to determine the optimized proniosomal formulation.

### Characterization of the developed vesicles

#### The effect of formulation variables on the EE% (Y_1_)

As shown in Table 1, the EE% of the prepared formulations ranged from 75.78 ± 0.61 (F3) to 85.45 ± 0.47% (F6). It can be concluded that the EE% significantly increased (*p* < 0.05) in all formulations prepared with Sp60 compared to Sp40 formulations, at the same drug amount and SAA:CH molar ratio. It can be also depicted that when SAA:CH molar ratio increased from 1:1 to 2:1, a decrease in EE % was seen with all prepared formulations. This might be attributed to the effect of higher CH content in the molar ratio SAA:CH 1:1, where the bilayer hydrophobicity and stability increase and permeability decreases [12]. This would improve the entrapment efficacy of hydrophilic drugs via stabilization of the bilayer membrane and inhibition of drug escape from the aqueous core assemblies (*p* < 0.05). A significant increase in EE % can be noticed in all formulations upon using 20 mg TZN instead of 10 mg (*p* < 0.05). This could be due to the fact that the medium is saturated with TZN, forcing the drug to be encapsulated in the vesicular system. Similar results were previously reported [45, 46].

#### Statistical evaluation of formulation variables on EE% (Y_1_)

**Fig. 1:**
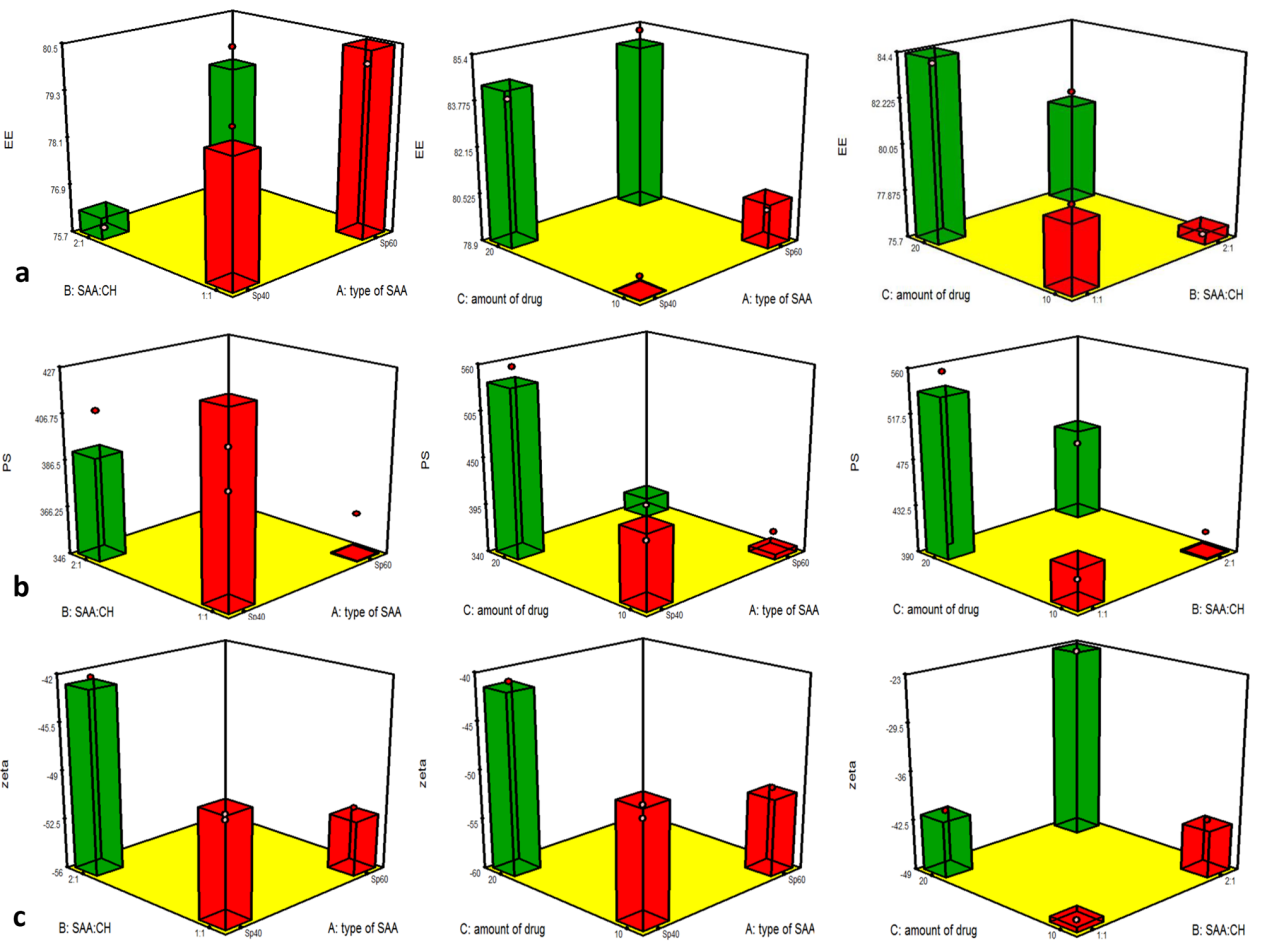
Graphical illustration for the factors interaction influence on **a** EE %, **b** PS, and **c** ZP, (*Y*_1_), **a** (*X*_1_,*X*_2_), **b** (*X*_1_,*X*_3_), and **c** (*X*_2_,*X*_3_)

As shown in Table 2, the model *F*-values of 31.71 imply the significance of the model with a correlation coefficient (*R*^2^) = 0.944 indicating for a good fit model. From the ANOVA statistical assessment, results showed that the effects of all single factors, *X*_1_, *X*_2_, and *X*_3_ on *Y*_1_ are statistically significant (*p* < 0.05), with *p*-values of 0.0004, 0.0017, and < 0.0001, respectively. On the contrary, the interaction terms (*X*_1_,*X*_2_), (*X*_1_,*X*_3_), and (*X*_2_,*X*_3_) showed non-significant effects on *Y*_1_ (*p* > 0.05), where the determined *p*-values were 0.499, 0.149, and 0.616, respectively. Figure 1a graphically represents the factor interactions on the dependent responses.

**Table 2:** ANOVA analysis of the dependent responses

**Responses**	**SS**	**MS**	**F-value**	***P*-value**	***R***^**2**^	**Adj. *R***^**2**^	**Pred. *R***^**2**^
**EE %**	105.58	15.08	19.34	< 0.0001	0.944	0.895	0.882
**PS**	72629.77	10375	382.11	< 0.0001	0.997	0.0.994	0.988
**ZP**	1453.25	207.60	88.14	0.0002	0.987	0.976	0.948

*SS* sum of square, *MS* mean sum of square, *R*^*2*^ determination coefficient, *Adj. R*^*2*^ adjusted determination coefficient, *Pred. R*^*2*^ predicted determination coefficient

#### The effect of formulation variables on PS (Y_2_)

The results show that the size of the prepared vesicles ranged from 348.22 ± 4.27 (F6) up to 559.82 ± 3.57 *n* (F5) (Table 1). As shown, the type of surfactant significantly influenced the particle size of the prepared proniosomes. Data in Table 1 generally represent a significant decrease in particle size with sp60 compared to sp40-proniosomes (*p* < 0.05). This might be attributed to the lower HLB value of Sp60 (4.7) compared to Sp40 (6.7), which made them smaller in size when both of them are compared at same drug amount or SAA:CH ratio. Similar results were reported previously [47]. Moreover, it can be concluded that the impact of cholesterol ratio on the size of niosomes was markedly significant, where a significant decrease (*p* < 0.05) in particle size was detected when the CH ratio decreased with both types of SAA when compared at same drug amount with the exception only for F8 which acquired bigger size when the CH ratio decreased. Cholesterol widens the bilayers, thus leads to an increase of the vesicular size [48, 49]. Regarding the drug amount, it can be observed, irrespective of surfactant type, that the particle size increased with increasing drug amount (*p* < 0.05), except for (F6). PDI values of the developed formulations were < 0.5 indicating homogeneity and narrow distribution of dispersed particles [11, 50, 51].

#### Statistical evaluation of the formulation variables on PS (Y_2_)

Along with the data represented in Table 2, the model *F*-values of 382.11 suggest the significance of the model with a correlation coefficient (*R*^2^) = 0.997 signifying for a good fit model. From the ANOVA test, outcomes exposed that the impact of all single factors, *X*_1_, *X*_2_, and *X*_3_ on *Y*_2_ are statistically significant (*p* < 0.05), with *p*-values of < 0.0001, < 0.0001, and < 0.0001, respectively. In addition, the interaction terms (*X*_1_,*X*_2_), (*X*_1_,*X*_3_), and (*X*_2_,*X*_3_) showed also a significant effect on *Y*_1_ (*p* < 0.05), where the determined *p*-values were < 0.0001, < 0.0001, and 0.0014, respectively (Fig. 1b).

#### The effect of formulation variables on ZP (Y_3_)

ZP is a vital indicator for dispersion stability [52]. The magnitude of ZP designates the degree of electrostatic repulsion between similarly charged particles in the produced formulation. When ZP values are ( ≤|20| mV), attractive forces can overcome repulsion forces causing accumulation. While at high ZP ( ≥|30| mV), the developed preparation is stable as high ZP values prevent aggregation and particles coalescence [52, 53]. All the developed formulations acquired a negative charge, ranged from −26.47 ± 3.92 to −59.64 ± 0.33 confirming good stable vesicles dispersion (Table 1). Upon studying the influence of SAA type on the ZP, we can see a significant higher ZP magnitude with Sp60 proniosomes compared with Sp40 proniosomes when compared at the same SAA:CH molar ratio and same drug amount (*p* < 0.05). The decrease in CH ratio generally decreased the ZP value with all formulations except F4 (*p* < 0.05). The reason behind that might be due to the increased viscosity of the bilayer membrane with increasing CH ratio, by preventing the surfactant bilayer’s gel-to-liquid phase transition, resulting in a more stable dispersion with a higher ZP magnitude [54].

#### Statistical evaluation of the formulation variables on ZP (Y_3_)

In conjunction with data displayed in Table 2, the model *F*-values of 88.14 propose the significance of the model with a correlation coefficient (*R*^2^) = 0.987 signifying for a good fit model. From the ANOVA test, outcomes exposed that the impact of all single factors, *X*_1_, *X*_2_, and *X*_3_ on *Y*_2_ are statistically significant (*p* < 0.05), with *p*-values of < 0.0001, < 0.0001, and < 0.0001, respectively. Furthermore, the interaction terms (*X*_1_,*X*_2_), (*X*_1_,*X*_3_), and (*X*_2_,*X*_3_) showed also a significant effect on *Y*_1_ (*p* < 0.05) where the determined *p*-values were 0.0024, < 0.0001, and < 0.0001, respectively, as shown in Fig. 1c.

### Optimization of TZN-PN

**Fig. 2:**
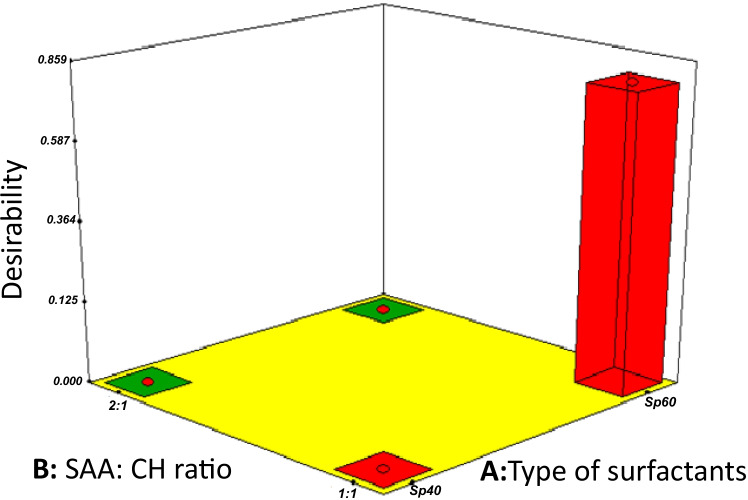
Graphical illustration for the desirability optimization for F6 (Sp60, 1:1, 20 mg TZN)

For the aim of producing an optimum proniosomal formulation under a given set of constraints, numerical and graphical optimization for the influence of all single factors as well as their interaction on the chosen responses was executed. The results, demonstrated in Fig. 2, came in favor for F6 composed of Span 60 as a surfactant type, with a surfactant:cholesterol ratio 1:1 and a TZN amount of 20 mg with a desirability range approaching 1, confirming the optimum results.

### Characterization of optimized TZN-PN

#### In vitro release studies

**Fig. 3:**
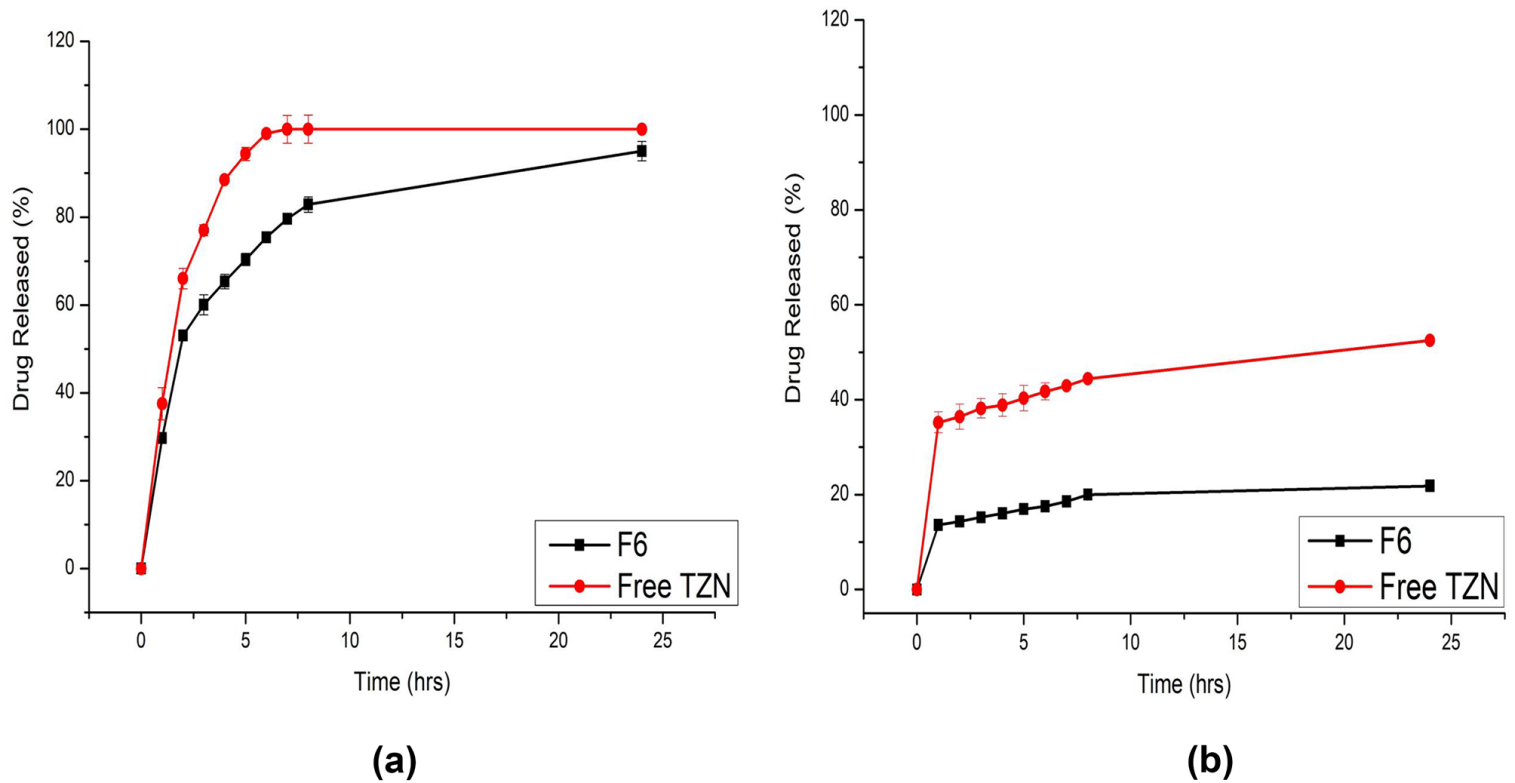
In vitro release profiles of drug from TZN-PN optimized formulation (F6) and free TZN at **a** 0.1 N HCL and **b** PBS 7.4

The release profile of free TZN, as well as optimized TZN-PN vesicular dispersions, in both gastric (pH1.2) and intestinal (pH 7.4) media is shown in Fig. 3. For both media, the drug released percentage from optimized TZN-PN vesicular system was lower than that released from TZN plain dispersion. TZN-PN exhibited a biphasic release profile that started by fast drug release which might be due to the initial release of drug particles attached to the surface of the vesicles. This was followed by a continuous slow release. At pH 1.2, the cumulative drug released percentage after 24 h was 95% and 100% for optimized TZN-PN formulation and free TZN, respectively. For pH 7.4, 21.84% and 52.5% were released from TZN-PN and free drug, respectively, after 24 h. Thus, it could be concluded that the drug released at acidic pH was higher as compared to its release at pH 7.4. This might be attributed to the effect of pH on TZN release. Similar results were previously reported for the in vitro release studies of cyclosporine [35] from niosomal vesicles. The vesicular carriers’ characteristics may be responsible for the drug’s long-term release. These vesicular carriers have the advantage of acting as drug reservoirs, allowing the encapsulated drug to release slowly. Similar results were reported for the release profiles of drugs from provesicular systems [25].

#### Kinetics study of the release profiles

Table 3 shows the release kinetics analysis of TZN from the hydrated vesicular formulations. The release data revealed no adequate fitting to zero order, first order, or Higuchi kinetics. Analyzing the release data with the Peppas model and explaining the release exponents “*n*” helps to clarify the mechanism that controls the release. As a result, the Peppas model was employed, where good linearity (*R*^2^ = 0.944 (PBS 7.4) and *R*^2^ = 0.8928 (0.1 N HCL)) was revealed (Table 3).

**Table 3:** The calculated correlation coefficients and kinetics parameters of TZN release profiles from the developed vesicular system

**Code**	**Zero order**	**First order**	**Higuchi**	**Peppas**	
	***R***^**2**^			***R***^**2**^	***n***
F6 (0.1 N HCL)	0.5320	0.3805	0.7005	0.8928	0.1959
F6 (PBS 7.4)	0.5982	0.5571	0.7644	0.9441	0.1667

According to Peppas’ hypothesis, drug release follows the Fickian diffusion mechanism if *n* ≤ 0.43, anomalous (non-Fickian) diffusion if 0.43 < *n* < 0.85, case II transport if *n* = 0.85, and super-case II transport if *n* > 0.85 [38]. The release exponent “*n*” value for optimized TZN-PN was 0.1667 and 0.1959 in PBS 7.4 and 0.1 N HCL, respectively, indicating a Fickian diffusion release mechanism. This comes in accordance with previously reported studies [55, 56], where the release of drug from different vesicular systems followed Fickian diffusion mechanism.

#### Transmission electron microscopy

**Fig. 4:**
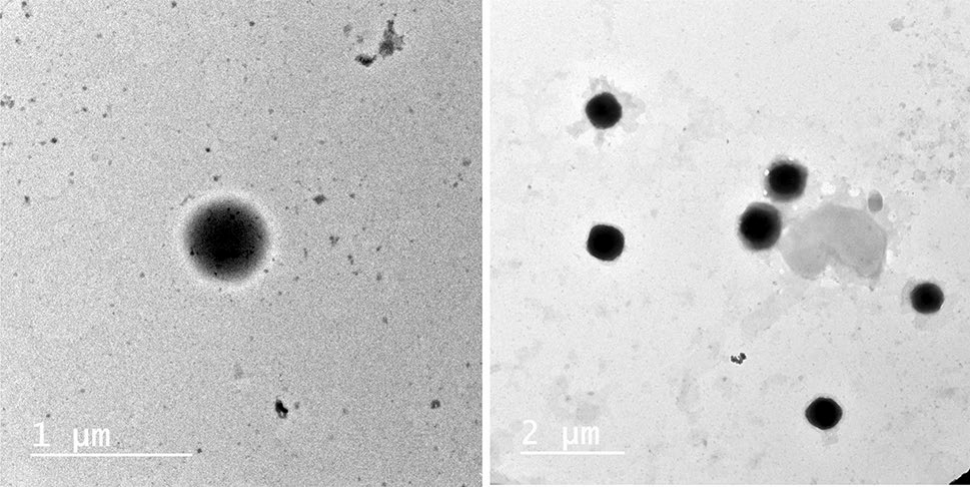
TEM micrographs of TZN-PN optimized formulation (F6)

TEM was employed to examine the morphology of the provesicular carriers after they had been hydrated for the optimized formulation F6. As demonstrated in Fig. 4, the vesicles are easily recognized as dark stained spherical structures with a smooth vesicular surface.

### In vivo studies

#### Effect of TZN-PN on muscle relaxant activity

**Fig. 5:**
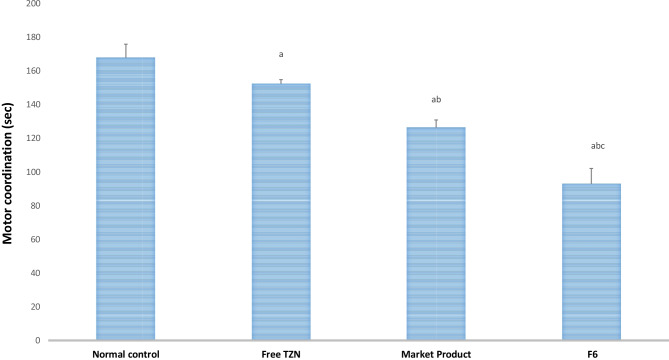
In vivo muscle coordination rotarod test performed on rats after oral administration of free TZN, marketed product and optimized TZN-PN. Data are presented as mean ± S.D. (*n* = 8). **a** Statistically significant different from normal control group at *p* < 0.05. **b** Statistically significant different from free drug group at *p* < 0.05. **c** Statistically significant different from market group at *p* < 0.05

Rotarod test (motor coordination) was employed for muscle relaxant assessment in animals [57]. Rats were given time on the revolving rod; less time on the rod showed a muscle relaxant effect. The effect of free TZN, market product, and optimized formulation on rat’s coordination is illustrated in Fig. 5. Treatment with free TZN, market product, and optimized formulation elevated coordination by 9%, 25%, and 45%, respectively, as compared to normal control, after 60 min of oral drug administration with significant difference (*p* < 0.05). As previously reported, TZN can significantly reduce muscle tone and also increase motor strength [58]. The elevation in coordination during the whole study can be arranged in the following order: normal control < free TZN < market product < TZN proniosomes (F6). In addition, treatment with optimized TZN formulation elevated rat’s coordination by 26% with comparison to the market product group. This influences the potential of the developed formulation in enhancing the therapeutic activity of TZN.

#### Effect of TZN-PN on GABA and EAAT2 levels

**Fig. 6:**
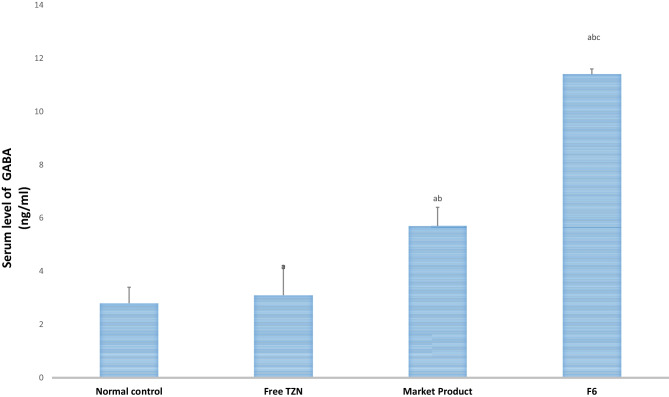
Gamma aminobutyric acid (GABA) serum levels after oral administration of free TZN, marketed product, and optimized TZN-PN. Data are presented as mean ± S.D. (*n* = 8). **a** Statistically significant different from normal control group at *p* < 0.05. **b** Statistically significant different from free drug group at *p* < 0.05. **c** Statistically significant different from market group at *p* < 0.05

Researchers believed that muscle-relaxant-like effect of drugs is related to its interference with GABAA [59], which is a chemical present in the brain and has anti-seizure and anti-anxiety effects. Thus, when muscle relaxant drugs elevate the levels of GABA, this induces muscle relaxation, sedation, and decrease in pain sensation [60]. It is known that TZN is a central α_2_ adrenergic agonist that can bind to GABAA receptor, inducing modification of the receptor structure and elevating GABAA receptor activity [61]. The results, demonstrated in Fig. 6, revealed that Free TZN, market product, and optimized TZN-PN displayed a significant (*p* < 0.05) elevation in GABA level by 11%, 104%, and 307% as compared to normal control. Furthermore, the optimized TZN-PN was able to double the level of GABAA when compared with market product (*p* < 0.05).

Glutamate is known as the primary excitatory amino acid found in the central nervous system. It is an excitatory neurotransmitter that is involved in nociceptive responses which are mediated by the activation of *N*-methyl-*D*-aspartate (NMDA), α-amino-3-hydroxyl-5-methyl-isoxazolepropionate (non-NMDA) receptors, and nitric oxide release. Nitric oxide enhances the release of the synthesis of pro-inflammatory mediators and reactive oxygen species (ROS) [62]. EAATs are the principal extracellular glutamate transporters in the neurological system. They not only have a remarkable ability to control ambient extracellular glutamate concentrations, but they can also control the temporal and spatial profiles of glutamate after vesicular release [63].

**Fig. 7:**
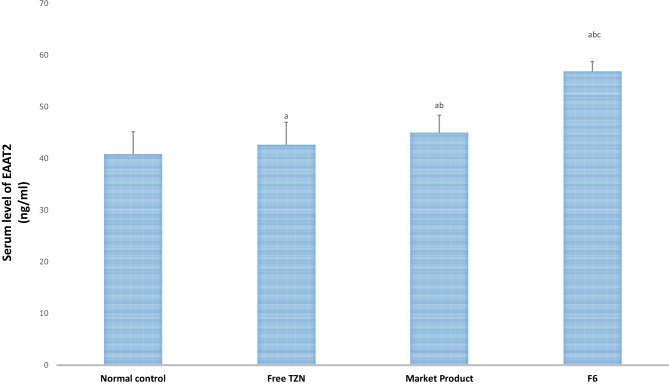
Excitatory amino acid transporter 2 (EAAT2) serum levels after oral administration of free TZN, marketed product, and optimized TZN-PN. Data are presented as mean ± S.D. (*n* = 8). **a** Statistically significant different from normal control group at *p* < 0.05. **b** Statistically significant different from free drug group at *p* < 0.05. **c** Statistically significant different from market group at *p* < 0.05

Excitatory amino acid transporter 2 **(**EAAT2) is responsible of transport of glutamate, thus increasing its levels leads to enhanced neurotransmission of glutamate [64]. The results, demonstrated in Fig. 7, revealed that free TZN, market product, and optimized TZN-PN formulation (F6) exhibited a significant (*p* < 0.05) elevation in glutamate transporter EAAT2 level by 4%, 10%, and 39% as compared to normal control. It was also observed that the optimized TZN-PN formulation (F6) increased EAAT2 level by 26% when compared with the market product group.

TZN is recognized as an a_2_-adrenoceptor agonist that have myotonolytic and antinociceptive activities at spinal and supraspinal levels [65]. It inhibits tonic stretch reflexes and polysynaptic reflex activity, potentially reducing nociception [66]. The obtained results suggested that encapsulation of TZN in proniosomes has enhanced the anti-nociceptive effect of the drug and consequently improved its therapeutic activity. Previous studies showed the efficiency of proniosomes in encapsulating various types of drugs with enhancement in their pharmacological activities [19].

## Conclusions

Tizanidine-loaded proniosomes (TZN-PN) were prepared employing coacervation phase separation method. A 2^3^ full factorial design was employed to attain an optimized TZN-PN formulation. The prepared TZN-PN showed high entrapment efficiency percentages (75.78–85.45%), vesicular sizes ranging between 348.22 and 559.82 nm, and zeta potential values ≤ −26.47. In vitro release profiles showed sustained release of TZN from the optimized proniosomal formulation, up to 24 h, when compared to free drug. In vivo studies revealed that the elevation in coordination throughout the experiment was in the order normal control < free TZN < market product < optimized TZN-PN (F6). Furthermore, the optimized TZN-PN showed significant muscle relaxant activity by 39% and 26% compared to control group and market product group, respectively. This was accompanied by an elevation in both GABA and EAAT2 serum levels. These findings reveal the potential of proniosomes in enhancing the muscle relaxant effect of TZN via the oral route.

## Data Availability

All data generated or analyzed during this study are included in this published article.
